# Iatrogenic biological fracture of the cervical spine during gradual halo traction for kyphotic deformity correction: case report

**DOI:** 10.1186/s12891-020-03350-x

**Published:** 2020-05-21

**Authors:** Austin Samuel Laifun Lim, Azizul Akram Bin Sali, Jason Pui Yin Cheung

**Affiliations:** Department of Orthopaedics and Traumatology, The University of Hong Kong, Professorial Block, 5th Floor, 102 Pokfulam Road, Pokfulam, Hong Kong SAR, China

**Keywords:** Cervical kyphosis, Halo traction, Iatrogenic fracture, Deformity correction, Osteotomy, Case report

## Abstract

**Background:**

Severe kyphotic deformities carry high risk for neurological injuries as osteotomies are often required for correction. Surgeons often utilize a staged approach for dealing with these conditions starting with a period of halo traction to stretch tight soft tissues and partially correct the deformity, followed by surgery. Halo traction is a relatively safe procedure and complications are uncommon. We report a unique case of iatrogenic fracture of the cervical spine during gradual halo traction for deformity correction of a severe cervical kyphosis.

**Case presentation:**

An 80-year-old female with previous cervical spine tuberculosis infection and C5-C6 anterior spinal fusion developed severe cervical kyphosis of 64° from C2-C6 and neck pain requiring deformity correction surgery. Gradual increase in traction weight was applied, aiming for a maximum traction weight of 45 pounds or half body weight. During the 1st stage halo-gravity traction, sudden neck pain and a loud cracking sound was witnessed during increase of the traction weight to 14 pounds. Imaging revealed a fracture through the C4 and reduction in kyphosis deformity to 11° from C2-C6. There was no neurological deficit. No further traction was applied and the patient underwent an in-situ occipital to T3 fusion without osteotomies. At 3-year follow-up, the patient was symptom-free and radiographs showed solid fusion and maintenance of alignment.

**Conclusions:**

Iatrogenic fracture may occur with halo traction. Elderly patients with osteoporotic and diseased bone should be closely monitored during the treatment. A fracture without complications was a fortunate complication as the patient was able to avoid any high-risk osteotomies for deformity correction.

**Level of evidence:**

IV

## Background

Kyphotic deformities of the cervical spine are rare but can cause debilitating disease due to axial neck pain and cord compression. Surgical intervention can correct the kyphotic deformity, restore lordosis and decompress the cord. One technique to achieve this is through posterior osteotomies, but they are technically demanding with high risk of complications. One study suggests an incidence of 13% for neurologic injury [1]. To minimize surgical risk, staged procedure with halo gravity traction followed by osteotomy is often adopted. The period of traction can stretch any tight neck muscles and partially correct the deformity. Less aggressive osteotomies can be performed at the second stage to reduce risk of complications. Comparatively, halo traction has less risk with mainly pin loosening (36%) and pin site infections (20%) as the predominant complications [2]. This is a case of an iatrogenic fracture of the cervical vertebrae while under halo gravity traction with an unintended result of correcting the cervical kyphosis to an alignment satisfactory for in-situ fusion without osteotomy.

## Case report

**Fig. 1:**
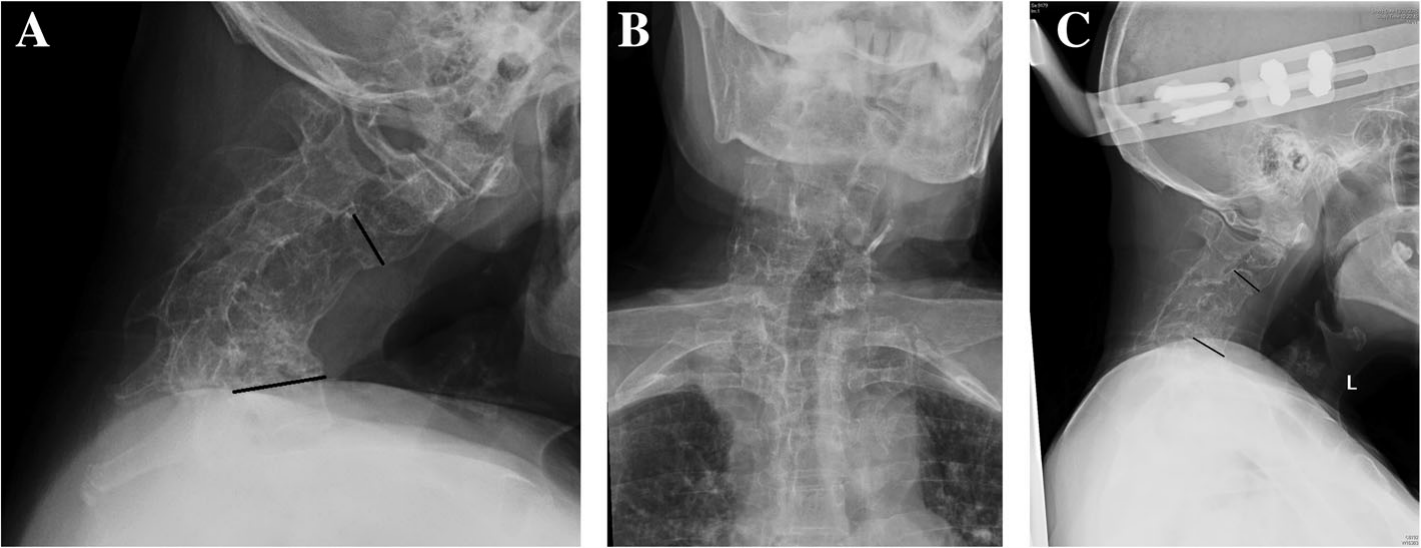
**a** Lateral cervical X-ray shows severe kyphotic angle of 64° before traction; **b** anteroposterior cervical X-ray shows a tilt to the left. **c** After the iatrogenic fracture at C4, the kyphotic angle improved to 11.2°

An 80 years old female with previous cervical spine tuberculosis infection, and underwent C5-C6 anterior spinal fusion in 1994, presented with severe cervical kyphosis. She also had comorbidities of dermatomyositis on azathioprine for more than 20 years. She had severe neck pain, and easy fatigue due to difficulties in maintaining a horizontal gaze. She did not have any complaints of hand clumsiness, paresthesia or radicular pain, and had intact hand function. However, examination identified bilateral hand weakness with motor power of 4/5, positive reverse supinator jerks and Hoffman’s sign. Cervical spine x-ray (Fig. 1a) revealed severe kyphotic deformity of 64° from C2-C6 with apex of the deformity at C5. There was a fused C4-C6 with osteoporotic bone. The head was also tilted to the left (Fig. 1b).

**Fig. 2:**
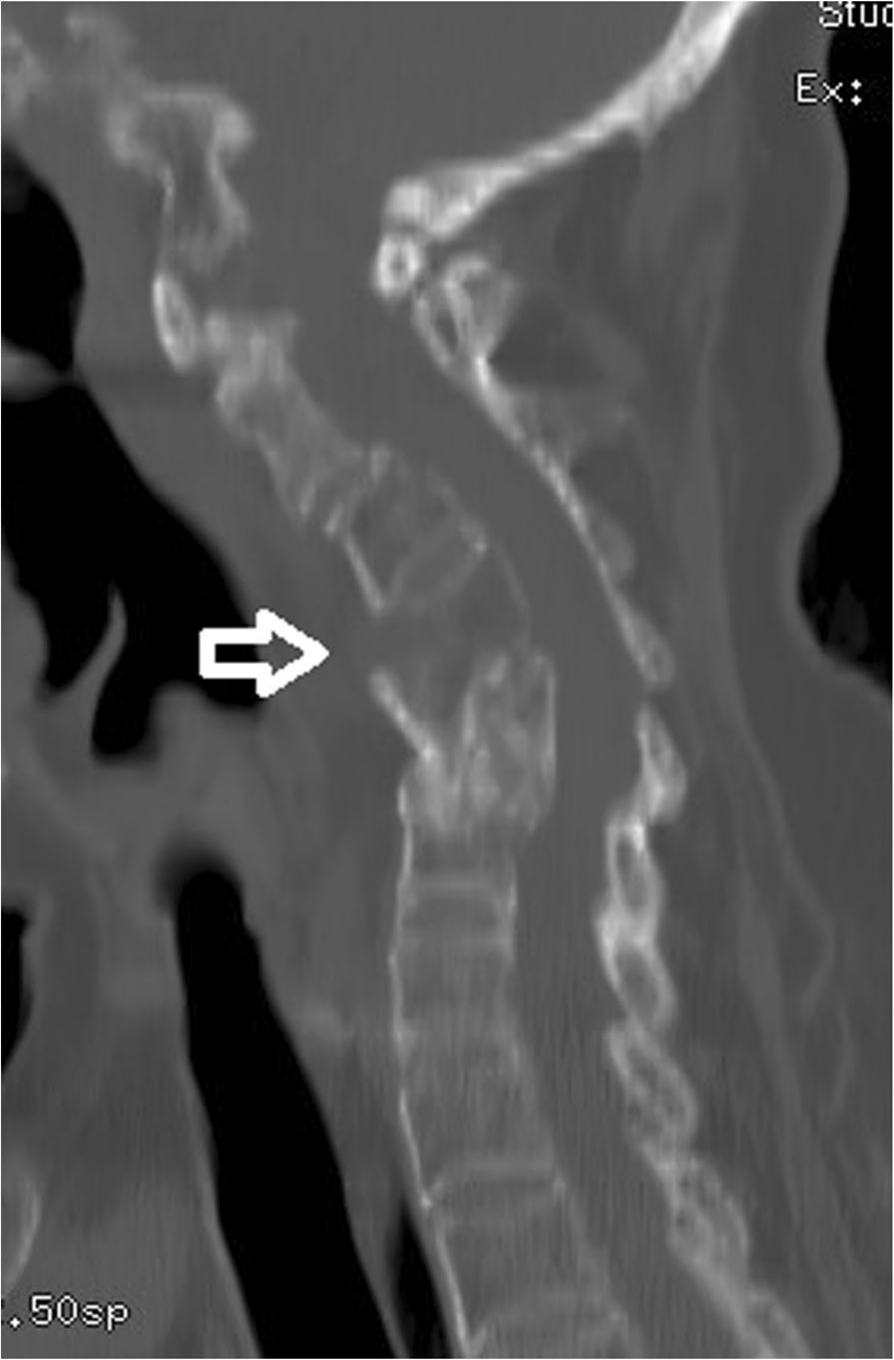
Sagittal CT scan after fracture with the white arrow pointing at the fracture line

**Fig. 3:**
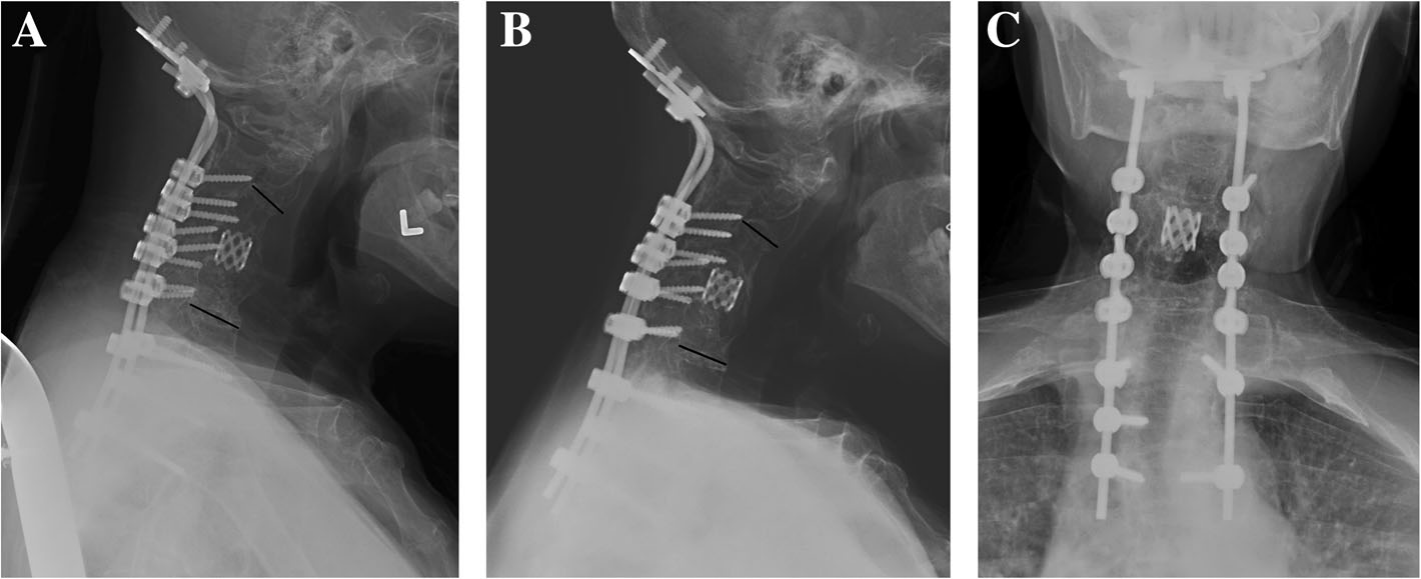
**a** Lateral cervical X-ray taken immediately postop showing a kyphotic angle of 15.4° from C2–6; **b** The kyphotic angle maintained at three-years after the anterior and posterior in-situ fusion and instrumentation with solid fusion; **c** The anteroposterior alignment was improved

A two-stage procedure involving halo-gravity traction followed by combined anterior and posterior instrumented fusion and multiple posterior column osteotomies was planned for her deformity correction. Since she weighed 110 pounds, maximum traction weight of 45 pounds was planned. Five pounds of traction was added after halo insertion followed by daily gradual increments of two pounds. The patient tolerated traction with no pin loosening nor cranial nerve palsies. At 14 pounds of traction weight, the patient complained of sudden neck pain and a loud cracking sound. X–ray taken immediately (Fig. 1c) with CT scan (Fig. 2). A fracture through the previously fused C4 vertebral body was found. There was no neurological compromise but the neck posture markedly improved. A reduction in kyphotic deformity was observed with good horizontal gaze. The kyphotic angle was measured to be 11° from C2-C6. No further traction was applied and she then underwent a combined anterior and posterior in-situ fusion without any osteotomies due to the satisfactory cervical alignment. An anterior cage was placed at C4-C5 with posterior iliac crest autograft. Posterior instrumented fusion was performed from occiput to T3 using an occiput plate with lateral mass screws from C3-C6 and pedicle screws from T1-T3 (Fig. 3a). The patient was well perioperatively and used a cervical collar for 2 weeks. She was then transferred to a rehabilitation centre on postoperative day 9 and discharged 2 weeks after surgery. At 3-years follow-up, she was symptom-free with solid fusion and maintenance of kyphotic correction (Fig. 3b) and head tilt (Fig. 3c).

## Discussion and conclusions

Surgical management of severe kyphosis usually requires multiple osteotomies. These procedures carry high complication rates (26.9–87.5%) with a mortality rate of 2.6% [3]. Complications include epidural bleeding, neurological impingement, C8 nerve palsy, non-union and infections [4], dysphagia and need for tracheostomy/intubation [5, 6]. Complications are more common for elderly patients, American Society of Anesthesiologists (ASA) classification of III or IV, and increased operative times [7]. Among these complications, cardiopulmonary dysfunction is the most common cause for mortality [8]. Patients undergoing subaxial spine osteotomies have more complications than patients undergoing higher thoracic osteotomies [1]. With the risks accompanying cervical osteotomy, one strategy to minimize this is to have the patient undergo gradual traction before the deformity correction surgery. Xiaojin et al reported an initial improvement of kyphotic deformity by approximately 20° with this method [9].

Complications with halo-gravity traction are uncommon but neurological deterioration including brachial plexus injuries and cranial nerve palsies may occur [10, 11]. Although there are a few reported cases of iatrogenic fracture during surgery for ankylosing spondylitis [12], this is the first report of an iatrogenic fracture to the cervical spine while on halo-gravity traction. Our patient has many risk factors leading to the iatrogenic cervical fracture including advanced age with a previously diseased bone and infection. The poor bone quality and tight soft tissues are important factors associated with the iatrogenic fracture. This is evidenced by the fracture occurring despite slow increase in traction at a relatively light weight. Nevertheless, it was a fortuitous complication as the patient did not develop any neurological compromise and avoided the high-risk osteotomies. In-situ fusion with an anterior cage was performed to encourage fusion across the anterior column defect at C4-C5.

This case serves to highlight that vertebral body fractures can occur during traction the cervical spine. Vigilant monitoring should be done for elderly patients with osteoporotic bone and with history of prior infection undergoing halo gravity traction.

## Data Availability

Not applicable as this is a case report.

## Abbreviations

ASA: American society of anesthesiologists
